# Deterioration Mechanism and Health Diagnosis Methods of Deep Anchoring Structures

**DOI:** 10.3390/ma19143131

**Published:** 2026-07-21

**Authors:** Shucan Lu, Saisai Wu, Moxuan Zhu, Krzysztof Skrzypkowski, Krzysztof Zagórski, Anna Zagórska

**Affiliations:** 1 Shaanxi Key Laboratory of Geotechnical and Underground Space Engineering, School of Resources Engineering, Xi’an University of Architecture & Technology, Xi’an 710055, China; 2 Faculty of Civil Engineering and Resource Management, AGH University of Krakow, Mickiewicza 30 Av., 30-059 Kraków, Poland; 3 Faculty of Mechanical Engineering and Robotics, AGH University of Krakow, Mickiewicza 30 Av., 30-059 Kraków, Poland; zagkrzys@agh.edu.pl; 4 Anna Zagórska-Research Centre in Kraków, Institute of Geological Sciences, Polish Academy of Science, Senacka 1, 31-002 Kraków, Poland; a.zagorska@ingpan.krakow.pl

**Keywords:** deep mines, anchoring structures, corrosion-induced deterioration, ultrasonic nondestructive testing, health diagnosis

## Abstract

As mineral resource extraction progressively extends to greater depths, the complex deep underground environment poses severe corrosion-induced deterioration risks to anchoring structures such as rock bolts. Anchorage failure has thus become a critical safety concern constraining the stability of deep roadways. To address the failure mechanisms of anchoring systems under multi-physical field coupling effects, this study conducts numerical simulations of multi-field corrosion processes and ultrasonic nondestructive testing (NDT) based on a numerical modeling platform. The influence of temperature on corrosion rate and current density is systematically analyzed, and interface response characteristics are extracted and interpreted for defects of varying dimensions. A spatial complementary mechanism under different corrosion defect configurations is revealed, and a health diagnosis system incorporating multiple critical indicators is established. The results indicate that elevated temperature significantly accelerates bolt corrosion: the rise in temperature shifts the equilibrium potential negatively and exponentially increases the reaction rate constant, both of which synergistically promote anodic dissolution. In ultrasonic testing, monitoring points along the main axis are positioned within the transmission-focused zone, where defects induce acoustic wave diffraction and superposition such that even minor defects cause a multiplication of the dominant frequency. Lateral monitoring points lie in the reflection–interference zone, where small defects preferentially attenuate energy, while larger defects manifest as amplitude reduction and first-arrival wave lag; all characteristic indices increase monotonically with defect size. Based on the numerical simulation outcomes, a four-level grading diagnosis standard and a “bottom–lateral” detection scheme are proposed as simulation-based reference indicators. The model effectively reproduces both corrosion deterioration and acoustic wave propagation characteristics, thereby providing a quantitative basis for the assessment of anchoring structures in high-temperature deep underground environments.

## 1. Introduction

With the progressive depletion of shallow mineral resources, mining depths have reached the kilometer level, giving rise to increasingly prominent “three-high” complex conditions—namely high in situ stress, high temperature, and high pore pressure—which pose severe challenges to the long-term stability of underground support structures [1,2]. As the primary supporting component for surrounding rock control in deep roadways, the service performance of rock bolts directly governs the overall safety of the roadway structure [3,4,5]. However, when long-term embedded in high-temperature mine water and subjected to complex in situ stress fields, rock bolts inevitably suffer from the synergistic effects of electrochemical corrosion and stress corrosion, leading to continuous deterioration of anchoring performance and, in severe cases, sudden safety incidents such as anchorage failure [6,7]. Therefore, an in-depth understanding of the corrosion deterioration mechanisms of rock bolts, along with the establishment of effective nondestructive testing methods, is essential for ensuring safe production in deep mines.

The deterioration of anchoring structures is highly concealed, as minor defects generated at an early stage are barely detectable by visual inspection or conventional methods. Such defects progressively reduce the load-bearing capacity of the anchoring system and may even trigger sudden failure, resulting in irreversible casualties and substantial property losses [8,9,10]. Although the conventional pull-out test can assess the bearing capacity of rock bolts, it is inherently destructive, introducing irreversible disturbances to the surrounding rock mass. Moreover, the test results exhibit a certain degree of randomness, which has progressively limited its applicability in engineering practice [11,12]. In contrast, ultrasonic nondestructive testing (NDT) technology, with its outstanding advantages of non-contact operation, non-destructive nature, high efficiency, and rapid implementation, is increasingly becoming the predominant technique for health monitoring of anchoring structures. It enables early identification and quantitative evaluation of anchoring defects, thereby providing reliable decision-making support for engineering maintenance [13,14,15]. Health diagnosis of anchoring structures is a comprehensive technical approach that involves three sequential steps: first, acquiring response signals via nondestructive testing means; second, extracting characteristic parameters; and finally, performing qualitative identification, localization analysis, and quantitative evaluation of defects—specifically determining whether defects exist, where they are located, and to what extent they have developed [16,17].

Significant breakthroughs have been achieved both domestically and internationally in nondestructive testing techniques for rock bolt anchorage quality. The United States took the lead in initiating technical research in the 1960s, establishing a detection method based on the stress wave reflection principle [18]. Subsequently, numerous countries including the United Kingdom, Germany, and Japan made sustained research efforts, leading to continuous refinement of this technology. Overseas researchers have developed several applicable nondestructive testing models from a theoretical perspective. American researchers constructed a wave propagation model for one-dimensional elastic rods, through which anchorage quality can be assessed by analyzing stress wave propagation characteristics. This work laid the theoretical foundation for the practical application of the reflection method in engineering [19]. British scholars focused on the interaction mechanism between rock bolts and the surrounding rock mass, striving to establish detection models that better reflect the actual working state of the surrounding rock, thereby consistently improving the reliability of test results [20]. Research teams at China University of Mining and Technology have conducted in-depth investigations into the propagation behavior of stress waves within rock bolts, systematically analyzing the disturbance mechanisms of anchoring defects on reflected wave signals [21,22]. Liu et al. [23], based on wavelet transform theory, developed a novel detection method capable of precisely extracting characteristic information from signals, significantly enhancing the accuracy and reliability of anchorage quality assessment and effectively addressing signal identification challenges in engineering practice. In recent years, ultrasonic nondestructive testing simulation techniques have attracted extensive attention [24]. Giurgiutiu et al. [25] and Shen et al. [26] employed piezoelectric wafer active sensors to conduct systematic theoretical and experimental studies on the propagation mechanisms of ultrasonic guided waves in rock bolts, clarifying the influences of geometric features and material properties on guided wave modes.

Although ultrasonic nondestructive testing (NDT) simulation techniques have achieved satisfactory results in defect localization in aerospace and industrial pipeline applications, their direct transferability to rock bolt anchoring structures is constrained by the heterogeneous interfaces involving mortar and surrounding rock within the anchorage system, which are not adequately represented by conventional wavefield models. To address this challenge, this study develops a numerical model integrating both corrosion deterioration and ultrasonic testing of anchoring structures based on a numerical simulation platform. The model couples electrochemical reactions with temperature field effects to quantify the accelerating influence of temperature on current density and corrosion rate. For defects of varying dimensions, ultrasonic simulations are employed to extract response characteristics and elucidate the spatial complementary mechanism. Furthermore, a multi-dimensional diagnostic index system is established, incorporating dominant frequency drift rate, echo time delay, and energy attenuation coefficient as core indicators, based on which grading criteria and a corresponding detection scheme are formulated. The findings of this research provide technical support for the theoretical framework of service life degradation and non-destructive monitoring of rock bolts.

## 2. Numerical Simulation of Deterioration and Nondestructive Testing of Rock Bolt Anchoring Structures

To address the durability deterioration of anchoring structures in deep mines, this study conducts an investigation into corrosion mechanisms and ultrasonic testing methods based on the COMSOL Multiphysics software (version 6.3). An electrochemical multi-physical field coupling model is established to investigate the accelerating influence of temperature on corrosion rate and its underlying mechanisms. Concurrently, an ultrasonic wave propagation model for rock bolts is developed to simulate the signal response characteristics under typical defect conditions.

### 2.1. Electrochemical Corrosion Multi-Physical Field Simulation

This study employs the secondary current distribution module of COMSOL Multiphysics to simulate and reproduce the electrochemical corrosion process of metallic rock bolts in soil environments, with a particular focus on analyzing the influence of temperature on corrosion rate and current density. EH36 high-strength structural steel was adopted as the baseline material for the rock bolts in the simulation. It should be noted that, although galvanized rock bolts are commonly employed in mining and tunneling engineering for corrosion protection, they were not considered in the present study. Instead, EH36 steel was selected because the primary objective of this work is to establish a fundamental electrochemical corrosion model for bare steel under temperature-varied conditions, thereby avoiding the additional complexity introduced by zinc coating kinetics and cathodic protection mechanisms, of which the primary electrochemical parameters are listed in Table 1.

In the present model, the “soil environment” is defined as the electrolyte solution domain filling the surrounding pores of the rock bolt. Its ionic conductivity is characterized by the measured electrical conductivity of mine water. The bolt surface is designated as an electrochemically active boundary, while the outer boundaries of the electrolyte domain are assigned as electrically insulating conditions to simulate the finite constraining effect of the surrounding rock/soil mass on the electric field distribution. In this study, a stress analysis is conducted on the rock bolt as the primary load-bearing component, whose stress corrosion behavior directly governs its long-term service performance. In the geometric modeling, the rock bolt is simplified as a cylinder with a diameter of 20 mm and a length of 1 m, with dimensions determined in reference to full-threaded rock bolts used in deep mines [27]. A free triangular mesh is adopted, with local refinement applied to the electrode surface region, where the maximum and minimum element sizes are set to 1 mm and 0.018 mm, respectively, to balance computational accuracy and solution efficiency. The FGMRES (Flexible Generalized Minimal Residual) solver is employed to handle the nonlinear coupling of multi-physical fields. Based on stationary analysis, temperature T is taken as the sweep parameter, with five temperature nodes (283 K, 293 K, 303 K, 313 K, and 323 K) selected at equal intervals over the range of 283–323 K. The distributions of electric potential, current density, and local corrosion rate on the bolt surface are solved at each temperature node.

COMSOL Multiphysics (version 6.2), employed in this study, is a finite element method-based multiphysics simulation platform, whose mathematical core lies in transforming partial differential equations into weak forms for discretized solution. The invoked “Secondary Current Distribution” interface physically solves the electric potential distribution within the electrolyte domain (governed by Ohm’s law) and couples it with the electrochemical kinetic equations at the electrode–electrolyte interface (i.e., the Tafel equations) to compute the local current density and corrosion potential of the electrochemical reactions. To accurately simulate the corrosion process of rock bolts in mine water, the following boundary conditions and coupling relationships are implemented in the model. The initial ionic potential in the electrolyte domain is set to zero, and the spatial distribution of electric potential is solved by Poisson’s equation. The electrolyte conductivity is assigned a value of 1.5 S·m^−1^, corresponding to the measured value in mine water. On the bolt surface, both anodic and cathodic Tafel equations are imposed, and the Butler–Volmer equation is employed to describe the unified electrode kinetic behavior [28], as given in Equation (1):

(1) $$i=i_{corr}\cdot [exp(\frac{ln10}{\beta_{a}}.\eta )-exp(\frac{ln10}{\beta_{c}}.\eta )]$$

where *i* is the measured current density; η (=*E* − *Ecorr*) is the overpotential, with *E* and *Ecorr* being the applied and corrosion potential; i_corr_ is the corrosion current density; and $\beta_{a}$ and $\beta_{c}$ are the anodic and cathodic Tafel slopes (V/dec), with $\beta_{c}$ being negative in this study.

The temperature field is introduced into the electrochemical equations as the primary sweep parameter. The temperature dependence of the corrosion thermodynamic driving force is implemented through the temperature derivative of the equilibrium potential, with (dφeq/dT = 0.54 mV/K). Concurrently, the temperature dependence of the electrolyte conductivity is calculated according to Equation (2) [29], accounting for the influence of temperature on ionic mass transfer rates.

(2) $$\sigma (T)=\sigma_{0}[1+0.020(T-T_{0})]\left(T_{0}=293K\right)$$

where $\sigma_{0}$ is the electrolyte conductivity at the reference temperature. The deformed electrode interface is coupled with the secondary current distribution module to update in real time the electrode surface morphology changes induced by metal dissolution, thereby incorporating the feedback effect of corrosion product accumulation on the subsequent current distribution. The distal end of the rock bolt is set as electrical ground (φ=0 V), which is consistent with the boundary condition encountered in practice where the bolt plate contacts the surrounding rock mass and conducts electricity.

**Table 1:** Electrochemical parameters of anchoring materials [30].

Parameter	Value	Unit	Measurement Condition
Electrical conductivity σ	4.032 × 10^6^	S/m	Ambient temperature and pressure
Equilibrium potential $\varphi_{eq}$	−0.44	V (vs. SHE)	Relative to standard hydrogen electrode
Temperature derivative of equilibrium potential	0.54	mV/K	Applicable in the range of 293–323 K
Anodic Tafel slope $\beta_{a}$	0.059	V/dec	-
Cathodic Tafel slope $\beta_{c}$	−0.118	V/dec	-
Exchange current density $i_{0}$	1.0 × 10^−6^	A/m^2^	Reference value, specifically at 293 K

### 2.2. Numerical Simulation of Wavefield Propagation for Nondestructive Testing

In practical anchoring engineering, rock bolts can be several meters in length and are tightly encased by mortar and surrounding rock. Conducting three-dimensional high-frequency ultrasonic simulations on full-scale structures would entail an excessively large mesh size, and the strong energy dissipation of the mortar layer would inevitably mask the scattering signatures of minor defects. To address this issue, the present study adopts a localized equivalent modeling strategy that focuses on the vicinity of defects. A three-dimensional material model with dimensions of 30 mm × 10 mm × 4 mm is established, and a cross-section is extracted after connecting the layered entities to perform two-dimensional analyses. The model material corresponds to the primary load-bearing material in the support system, which possesses high tensile and yield strengths capable of withstanding the in situ stresses and deformations of deep rock masses; the detailed material parameters are listed in Table 2. A sinusoidal wave is selected as the excitation signal owing to its favorable single-frequency characteristics, which enable clear visualization of wave propagation behavior at a specific frequency. Compared with the widely used pulse signals, the sinusoidal wave is less susceptible to waveform distortion or signal-to-noise ratio degradation caused by medium attenuation and boundary scattering, making it more suitable for the numerical simulations in this study with balanced accuracy and efficiency. The excitation source is applied at the midpoint of the top boundary, and monitoring points are arranged along both the axial and lateral directions. This simplified model effectively eliminates interference from complex external boundaries, allowing a focused analysis of the interaction between high-frequency ultrasound (3 MHz) and microscopic defects. It should be noted that the present simulation focuses on wave–defect interaction under the assumed conditions, and does not incorporate the complex mechanical stress states encountered in practice, which is left for future investigation.

Under electrochemical corrosion, the material undergoes progressive damage over time. At the macroscopic level, the local cross-section gradually becomes thinner, and under severe conditions, geometrically discontinuous regions may develop. This process can be equivalently described by the product of corrosion rate and exposure time, as expressed in Equation (3):

(3) D=v⋅T

where D is the equivalent defect characteristic size (mm), representing the geometric scale of corrosion-induced damage; T is the corresponding equivalent service time (a, years); and v is the corrosion rate, taken as 0.70 mm/a. Based on the above relationship, the corrosion extent under test conditions can be mapped to the geometric defect parameters in the numerical model, thereby enabling a quantitative transformation from experimental results to the simulation framework. The input parameters for the simulation, including the corrosion rate and electrochemical properties, are based on our prior experimental results [30].

**Figure 1:**
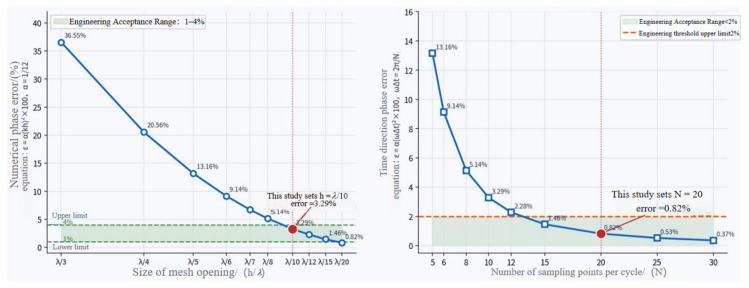
Convergence analysis diagram for grid size and time step parameters.

To balance the accuracy and computational efficiency of ultrasonic wave propagation simulations, this study evaluates the numerical phase error under different discretization conditions based on wave propagation theory, and the convergence curves are presented in Figure 1. The analysis results indicate that as the maximum mesh size mesh is progressively refined from λ/3, the spatial error decreases continuously. At mesh = λ/3 and λ/8, the errors are 36.55% and 5.14%, respectively, both exceeding the acceptable engineering tolerance. When refined to mesh = λ/10, the error reduces to 3.29%, falling within the permissible range of 1–4%. Further mesh refinement yields only marginal improvements in accuracy while substantially increasing computational cost. Therefore, mesh = λ/10 is selected as the maximum mesh size in this study. The temporal error as a function of the number of sampling points per cycle N shows that when N does not exceed 10, the error exceeds the engineering accuracy requirement of 2%. When N reaches 15 or higher, the error can be reduced to within 2%. To avoid distortion of high-frequency components, each period corresponding to the maximum analysis frequency is divided into 20 equal intervals; i.e., the time step parameter is set to N = 20, for which the corresponding temporal error is only 0.82%. The spatial mesh and time step adopted in this study both meet the accuracy requirements, satisfy the CFL (Courant–Friedrichs–Lewy) numerical stability condition, and achieve a favorable balance between accuracy and computational scale in the ultrasonic wavefield simulations. It should be noted that the present localized model isolates the wave–defect interaction and does not include the mortar/rock layers or interfaces; the quantitative indicators are therefore intended primarily for comparative assessment of defect severity.

### 2.3. Validation of the Ultrasonic Wave Propagation Model

**Figure 2:**
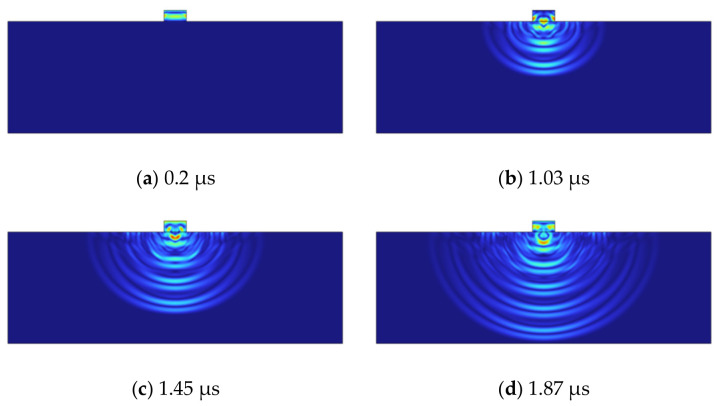
Sinusoidal wave propagation within the model.

Figure 2 illustrates the propagation process of a sinusoidal wave within the model at different time instants. The excitation wave emanates from the source and gradually diffuses into the material. When the wave encounters interfaces, partial reflection occurs, while the remaining energy transmits through and continues to propagate. The source is located on the symmetry axis of the model, and the wave energy is concentrated in the vicinity of the axis, with the amplitude decreasing with increasing off-axis distance. The wavefront remains planar, with energy well confined along the propagation direction, and no significant scattering or distortion is observed. The interference formed by the superposition of reflected and incident waves at specific time intervals confirms that the finite element model is capable of not only simulating the direct propagation of P-waves but also effectively capturing reflection phenomena. In summary, the simulation results demonstrate that the model can reasonably describe the propagation characteristics of ultrasonic waves in materials in both temporal and spatial domains.

To further verify the authenticity and accuracy of the finite element simulation results, this study calculates the theoretical propagation time delays segment by segment based on the geometric distances at the model interfaces (determined from geometry modeling) and compares them with the propagation time delays observed in the COMSOL simulations. The relative errors are then evaluated, and the sources of error are discussed. The relative error is defined as shown in Equation (4):

(4) $$\mathrm{error}(\%)=\frac{\left|\Delta t_{\mathrm{obs}}-T\right|}{T}\times 100\%$$

where Δt_obs_ is the propagation time delay observed in the simulation (μs), and T is the theoretical propagation time delay, calculated by T = d/v geom. Here, d is the geometric distance in meters, given by d = d geom/1000 (m), where dgeom is the geometric distance between adjacent interfaces in millimeters (mm).

To quantitatively evaluate the simulation accuracy, three adjacent segments are selected from the simulation results based on the acoustic waveform, and the propagation time delays of each segment are calculated and compared with the theoretical values. The geometric distances of the three interfaces are 6.10 mm, 7.28 mm, and 7.28 mm, corresponding to simulated time delays of 1.05 μs, 1.25 μs, and 1.25 μs, respectively, as summarized in Table 3. The data show that the theoretical and simulated time delays for the three segments are in good agreement, with all relative errors below the conventional engineering accuracy threshold of 2% and absolute deviations within 0.02 μs. These results demonstrate that the COMSOL model can accurately reproduce the propagation process of ultrasonic P-waves, and the simulation data are sufficiently reliable for subsequent defect localization and quantitative analysis.

## 3. Corrosion Deterioration Characteristics of Anchoring Structures and Ultrasonic Detection Results

### 3.1. Accelerating Effect of Temperature on Electrochemical Corrosion of Rock Bolts

**Figure 3:**
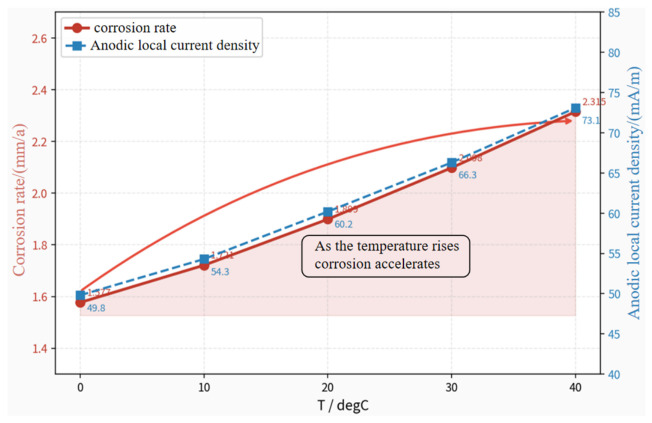
Variation in corrosion rate and local anodic current density with temperature.

Through steady-state parametric sweeping, this study investigates the effects of temperature (283 K–323 K) on the corrosion rate, surface potential distribution, and local current density distribution of rock bolts. As shown in Figure 3, the corrosion rate increases continuously with rising temperature, with a cumulative increment of approximately 46.8%. This phenomenon can be interpreted from both thermodynamic and kinetic perspectives. Thermodynamically, the rise in temperature shifts the equilibrium potential in the negative direction (0.54 mV/K), thereby increasing the reaction driving force and promoting anodic dissolution. Kinetically, the reaction rate constant k increases exponentially with temperature; an increase of 10 °C in temperature corresponds to an approximate 15–25% enhancement in the reaction rate. This trend is mechanistically consistent with the existing experimental results on atmospheric corrosion [30], thereby validating the adaptability of the electrochemical parameter settings in the present model to high-temperature underground environments. Meanwhile, the anodic local current density exhibits a synchronous increasing trend with the corrosion rate, with an increment of approximately 46.8%, further indicating that the temperature rise accelerates corrosion by intensifying anodic dissolution, without interference from other competing pathways.

**Figure 4:**
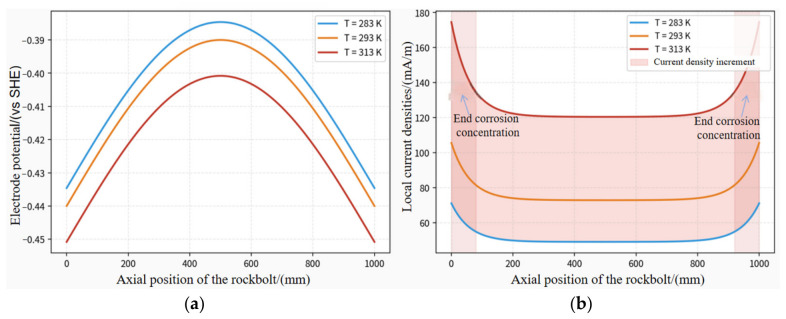
Axial potential and surface current density distribution of the rock bolt at different temperatures. (**a**) Axial potential distribution diagram of rock bolts at different temperatures. (**b**) Surface current density distribution diagram of rock bolts at different temperatures.

As shown in Figure 4a, the axial potential distribution along the rock bolt generally exhibits a “higher in the middle, lower at both ends” pattern. This is primarily attributed to the more thorough contact between the bolt ends and the solution, where the local potential becomes more negative, and the corrosion driving force is correspondingly stronger. This end effect arises because the insulating outer boundaries force current lines to converge toward the bolt ends, steepening the potential gradient and producing a more negative local potential, which in turn increases the activation overpotential and the corresponding corrosion driving force. When the temperature increases from 283 K to 313 K, the overall potential of the bolt shifts in the negative direction by 0.04 V. This shift is comparable in magnitude to the theoretical value estimated from the temperature derivative of the equilibrium potential. The negative shift of the overall potential indicates that the temperature rise thermodynamically promotes anodic dissolution, which is the fundamental cause of accelerated corrosion. As shown in Figure 4b, the current density distribution intuitively reveals the engineering reality of corrosion concentration at the bolt ends: the current densities at both ends are significantly higher than that in the middle section, forming a corrosion morphology characterized by end effects. Taking 293 K as the reference, the local current density in the end region of the bolt (z = 0–50 mm) is approximately 1.8 times that in the middle section, indicating that the two ends are the priority corrosion zones. This result is consistent with the findings from numerical simulations and provides a theoretical basis from a numerical perspective for the engineering practice of enhanced anti-corrosion treatment at the ends of anchoring structures. In summary, temperature exerts a significant positive accelerating effect on the electrochemical corrosion rate of rock bolts, which is the fundamental electrochemical cause of accelerated deterioration of anchoring structures in the high-temperature environment of deep mines. The bolt ends, due to the end effect, form localized corrosion concentration zones and are therefore the critical locations for engineering protection.

### 3.2. Ultrasonic Response Characteristics of Defects and Identification of Sensitive Parameters

#### 3.2.1. Signal Preprocessing and Definition of Characteristic Parameters

For the established ultrasonic propagation model, this study systematically performs signal feature extraction and analysis under various defect conditions. Through preprocessing of the simulated signals, the spatial complementary mechanism between the monitoring points on the main axis and those in the lateral direction is revealed, and the physical causes underlying the response differences between minor and major defects are elucidated. The signal acquisition parameters are as follows: a time window of 0–5 μs and a time step of 16.667 ns, yielding a total of 301 sampling points. The sampling frequency satisfies the Nyquist sampling theorem, effectively avoiding signal aliasing. The raw displacement signals directly reflect the intensity of ultrasonic wave motion. In the defect-free model, the signals exhibit no abnormal attenuation or time delay, whereas in the defective models, the peak arrival times and amplitudes of the displacement signals undergo significant changes due to the reflection and scattering effects of defects on acoustic waves, providing a data foundation for defect identification.

**Figure 5:**
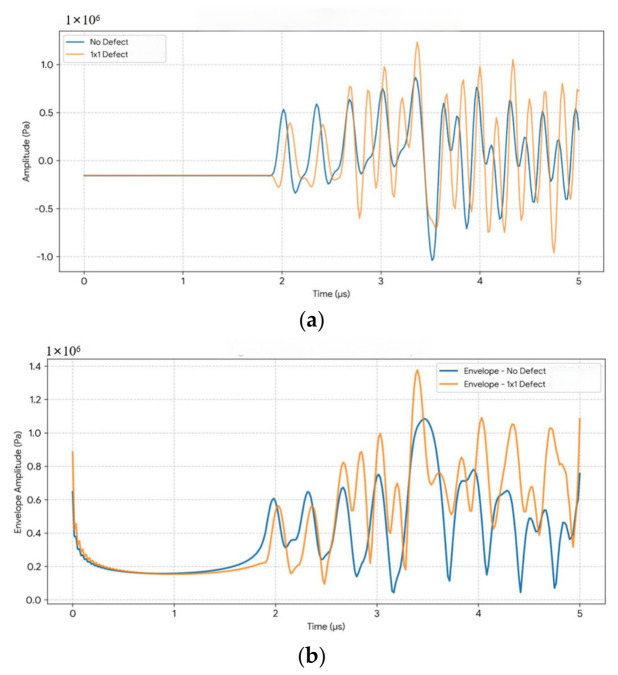

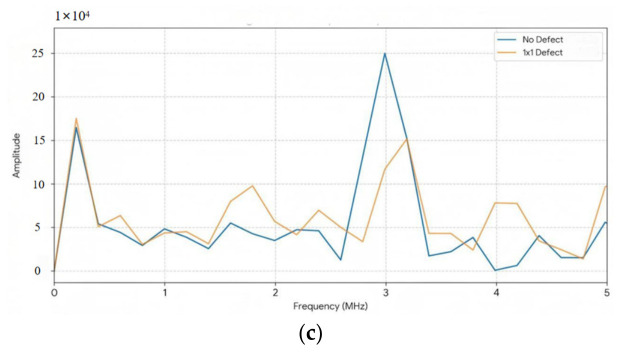
Signal processing result. (**a**) Time-domain signal graph. (**b**) Hilbert transform envelope plot. (**c**) FFT amplitude spectrum diagram.

The raw ultrasonic signals suffer from issues such as DC offset and high-frequency oscillatory interference, making direct analysis inadequate for accurate extraction of characteristic parameters. Therefore, a preprocessing workflow is necessary to eliminate interference and highlight the essential signal features. First, to remove the DC offset introduced by the COMSOL numerical calculations, a mean-subtraction preprocessing step is applied to avoid overestimation of the energy integral and to ensure the objectivity of subsequent analyses. On this basis, the Hilbert transform is performed on the preprocessed signals to extract the envelope curves. The envelope converts the high-frequency oscillatory waveform into a profile that reflects the temporal energy distribution of the acoustic wave, where the peak position corresponds to the wave arrival time, and the amplitude decay trend maps the scattering and absorption effects of defects on acoustic waves. By comparing the envelope peak delay and amplitude reduction, the geometric characteristics of the defects can be inversely inferred. Furthermore, the single-sided FFT based on time-step calibration is employed to obtain the true amplitude spectrum. The localized stiffness reduction induced by defects leads to a downward shift of the center frequency toward the low-frequency range, while the high-frequency components are attenuated due to scattering. Quantitative analysis of the frequency shift and attenuation rate elucidates the frequency-domain modulation mechanism of defects from the perspective of energy redistribution. The signal preprocessing results are presented in Figure 5.

To quantify the signal differences, four characteristic parameters are defined: propagation time delay, amplitude ratio, signal energy, and spectral energy distribution. Based on the previously established model, a controlled-variable approach is adopted for comparative investigations. A defect-free model is used as the baseline, against which three defective models with defect sizes of 1 mm × 1 mm, 2 mm × 2 mm, and 3 mm × 3 mm are established. These specific dimensions were determined based on the corrosion rate of 0.70 mm/a derived from our previous experiments [30], corresponding to equivalent service times of approximately 1.43, 2.86, and 4.29 years, respectively. This selection covers a representative range of corrosion-induced damage from early-stage local thinning to severe section loss. Furthermore, relative to the ultrasonic wavelength at 3 MHz (approximately 2.0 mm in steel), the selected sizes represent sub-wavelength, wavelength-scale, and super-wavelength regimes, which enables a systematic investigation of defect-size-dependent scattering mechanisms. Two representative monitoring points are selected: the main-axis point (15, 0), located on the incident-wave side (near-field region), and the lateral point (20, 10), situated on the wave-transmission side (far-field region), to capture the reflection enhancement and transmission attenuation characteristics induced by defects, respectively.

#### 3.2.2. Response Characteristics and Sensitivity Evaluation at the Main-Axis Monitoring Point

The main-axis monitoring point (15, 0), located in the near-field region on the incident-wave side, is primarily intended to capture the reflection enhancement characteristics at the defect interface. This point is situated on the central axis at the bottom of the model, directly beneath the ultrasonic source. Under normal expectations, this location would experience signal attenuation due to the shielding effect of the defect. However, the numerical simulations reveal that, under symmetric structural conditions, this monitoring point does not exhibit attenuation; instead, it displays wave convergence and amplitude amplification. As shown in Table 4, the acoustic pressure at the bottom of the main axis exhibits an anomalous enhancement, and the signal becomes stronger with increasing defect size. For the 1 mm and 2 mm defects, the main peak amplitudes are both 1.38 × 10^6^ Pa, representing a 27.8% increase over the defect-free case (1.08 × 10^6^ Pa), with the energy enhancement exceeding 50%. For the 3 mm defect, the amplitude rises sharply to 1.99 × 10^6^ Pa, and the energy reaches 5.2 times that of the defect-free model. It is evident that the defect alters the acoustic energy distribution at this specific location, forming a high-intensity convergence zone.

**Figure 6:**
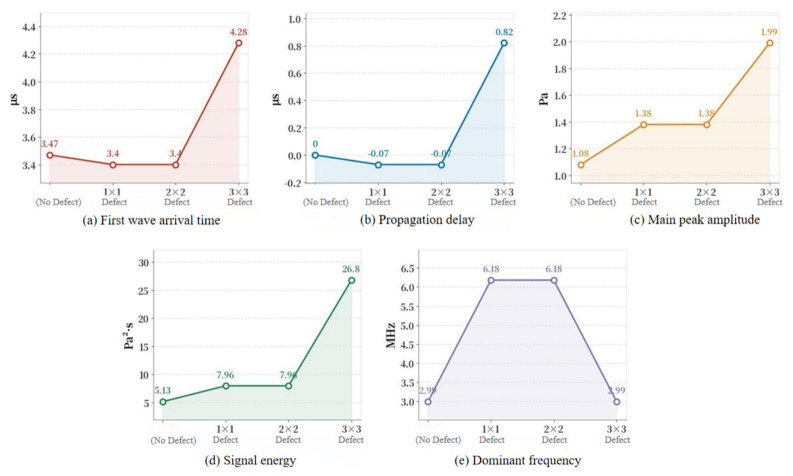
Comparison chart of ultrasonic signal characteristic parameters at the main axis line monitoring points under different defect sizes.

When the acoustic wave reaches the edges of the defect, diffraction occurs, and the superposition of multiple wave trains elevates the local signal intensity. As shown in Figure 6, the monitoring point (15, 0) is located on the structural symmetry axis. The defect blocks the propagation of the plane wave, forcing the acoustic wave to diffract around the two ends of the defect. The geometric symmetry ensures that the diffracted waves from both sides travel equal path lengths and arrive synchronously with an identical phase. According to the principle of wave superposition, the constructive interference of in-phase wave crests yields a resultant signal superior to that of the conventional transmitted signal. For the 3 mm defect, the direct wave is substantially blocked, and the received signal is predominantly formed by the superposition of high-energy diffracted waves. The diffraction path introduces a time delay of approximately 0.82 μs, separating the wave crest from the tail of the direct wave and producing an exceptionally high amplitude. Frequency-domain analysis reveals that the dominant frequencies of both the defect-free model and the 3 mm defect model remain stable at 2.99 MHz, whereas for the 1 mm and 2 mm minor defects, the dominant frequency is multiplied to 6.18 MHz. This phenomenon arises because, when the defect size is comparable to the wavelength, low-frequency long waves are readily obstructed or scattered, while high-frequency short waves continue to propagate via edge diffraction and superpose along the axis, resulting in a dominant frequency multiplication feature that can serve as a key quantitative indicator for identifying early-stage minor defects. In summary, the signal characteristics at the main-axis monitoring point (15, 0) are governed jointly by geometric symmetry and wave superposition effects, exhibiting high sensitivity to defects, manifested as a simultaneous increase in both amplitude and dominant frequency. This enhancement phenomenon is confined to the axial position; deviation from the axis reverts to conventional attenuation behavior. In engineering practice, the focusing characteristics at this point can be utilized for qualitative early warning, which can then be complemented with lateral monitoring points for comprehensive assessment.

#### 3.2.3. Response Characteristics and Sensitivity Evaluation at the Lateral Monitoring Point

Unlike the transmission focusing mechanism at the main-axis monitoring point, the lateral monitoring point (20, 10) is located in the lateral reflection zone on the upstream side of the defect, 5 mm to the right of the wave source. The signals at this point consist of both the direct wave and the scattered waves reflected upward from the defect interface. The temporal phase interference between these two wave trains governs the response characteristics at this location. The comparison of ultrasonic signal characteristic parameters under different defect sizes is summarized in Table 5.

**Figure 7:**
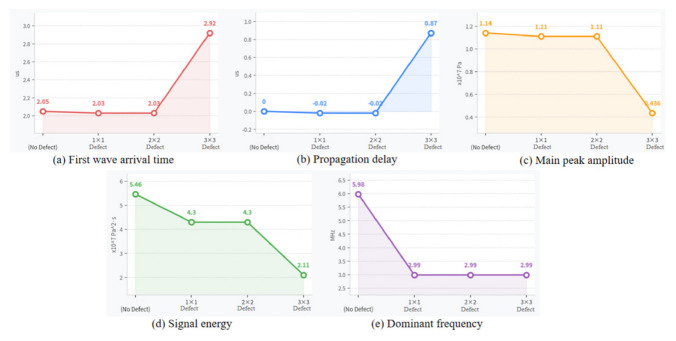
Comparison chart of ultrasonic signal characteristic parameters at different defect sizes of lateral monitoring points.

As shown in Figure 7, the quantitative data indicate that the signals at the lateral monitoring point result from the superposition and interference of the direct wave and the reflected wave. The smaller the defect, the weaker its influence; the larger the defect, the stronger its influence, even leading to phase-cancellation. For minor defects (1 mm and 2 mm), the reflection surface is too small, resulting in an extremely weak reflected wave, and the direct wave dominates overwhelmingly. The arrival time and amplitude of the first wave are highly consistent with those of the defect-free case. For the 3 mm defect, the arrival time of the first wave is delayed to 2.92 μs (a lag of 0.87 μs). This delay is not caused by path obstruction but rather by the out-of-phase superposition of the reflected wave and the direct wave, where the first peak is weakened or even annihilated, and the subsequent secondary peak is misidentified as the first wave, resulting in an apparent lag.

The energy response indicates that for the 1 mm and 2 mm minor defects, although the main peak amplitude decreases only slightly, the total signal energy drops from 5.46 × 10^7^ Pa^2^·s to 4.30 × 10^7^ Pa^2^·s, representing an attenuation of 21.2%. Although the weak reflected wave does not alter the waveform of the first wave, its superposition with the subsequent wave train leads to cancellation, resulting in a reduction in total energy. Therefore, when identifying early-stage minor defects, the energy indicator is more sensitive than the amplitude indicator. In terms of frequency-domain response, the dominant frequency of the signal decreases from 5.98 MHz–2.99 MHz in the presence of defects. This is because high-frequency waves, with their shorter wavelengths, are more susceptible to cancellation through superposition, while low-frequency waves with longer wavelengths are preserved, resulting in a substantial suppression of high-frequency components in the signals received at the lateral point. In summary, the lateral monitoring point is less sensitive to minor defects and is prone to missed detection, whereas the main-axis point is more sensitive. However, the lateral point responds dramatically to major defects, with a 60% amplitude reduction and a 0.87 μs time delay, making it suitable for identifying macroscopic fractures or severe damage. In practical applications, a substantial amplitude decrease accompanied by a significant delay of the first wave typically indicates the presence of extensive severe damage within the structure.

## 4. Health Diagnosis Method for Anchoring Structures Based on Sensitive Characteristic Parameters

### 4.1. Sensitive Characteristic Evaluation System and Spatial Complementary Mechanism

**Figure 8:**
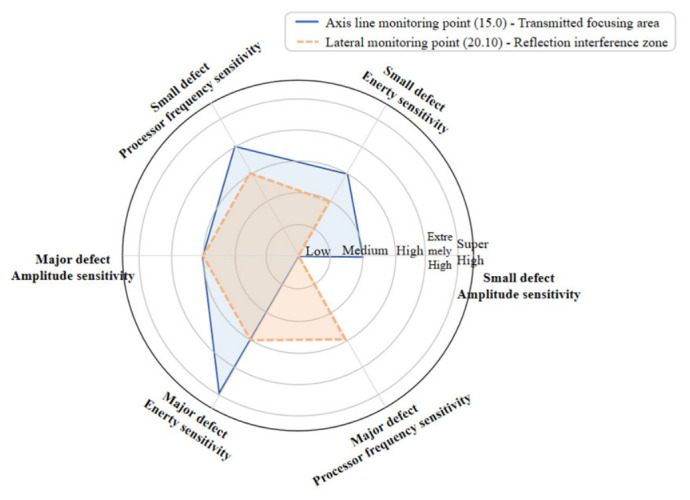
Radar chart illustrating sensitivity evaluation of defect characteristic parameters at different monitoring positions.

The ultrasonic response characteristics indicate that a single monitoring point or a single indicator is inadequate for simultaneously achieving both the detection of minor damage and the quantitative assessment of deterioration severity. To address this issue, this study performs a graded sensitivity evaluation of the characteristic indicators based on the response differences of detection waves along different propagation paths, and consequently establishes a health diagnosis strategy. Three core indicators—amplitude, energy, and dominant frequency—are selected to calculate their relative variations under minor defects (1–2 mm) and large defects (3 mm), and a sensitivity evaluation radar chart is constructed (Figure 8). The results demonstrate that the main-axis monitoring point exhibits significant responses in the dimensions of “minor defect–dominant frequency” and “large defect–energy”: minor defects induce dominant frequency multiplication, while large defects cause a surge in energy. Located in the transmission-focused zone, this point is sensitive to incipient defects, exhibiting stepwise signal changes, making it suitable as a qualitative early warning indicator for early-stage damage. The lateral monitoring point presents a relatively balanced distribution of characteristic features, with high sensitivity across all indicators under large defects, and outperforms the main-axis point in the “minor defect–energy” dimension in terms of amplitude indicators. Situated in the reflection–interference zone, the signal variations at this point are less drastic than those at the main-axis point, but the indicators increase monotonically with increasing defect size.

The numerical simulation results indicate that the monitoring signals at different spatial locations exhibit a high degree of complementarity in reflecting defects, providing a physical basis for multi-point joint diagnosis. The transmission-focused zone at (15, 0) achieves signal amplification through diffraction focusing, effectively highlighting the responses of minor defects and compensating for the susceptibility of conventional ultrasonic testing to miss early-stage fine cracks. However, signals at this point are prone to saturation, and its capacity to distinguish between defects of similar sizes, such as 1 mm and 2 mm, is limited. The reflection–interference zone at (20, 10), on the other hand, reflects the obstruction of the propagation channel through the superposition and cancellation of reflected and direct waves. Although it is less sensitive to waveform changes caused by early-stage minor defects, it can accurately identify the time delay and energy attenuation induced by large defects, thereby compensating for the limitations of the main-axis point in quantitative assessment during the severe damage stage. Based on the above sensitivity evaluation and complementary mechanism, Table 6 summarizes the response characteristics of each characteristic parameter at different damage stages and defines their respective functions in the graded diagnosis framework.

It should be emphasized that the diagnostic criteria and grading thresholds proposed in this study are derived from numerical simulation results and laboratory experiments. Although the simulation model has been validated against experimental data from the literature, the quantitative indicators—including the dominant frequency drift rate (R_f_ > 50%), energy attenuation coefficient (R_E_ > 50%), and echo time delay (Δt > 0.5 μs)—are simulation-based reference values. This evaluation framework is intended to provide a theoretical basis for assessing corrosion-induced defects in anchoring structures.

### 4.2. Diagnostic Index System and Grading Criteria

Based on the spatial complementary relationship between transmission focusing and reflection interference, and considering the differences in sensitivity of various characteristic parameters to defects, this paper proposes a graded evaluation scheme for nondestructive testing of anchoring structures in deep mines. This scheme fully integrates multi-dimensional characteristic information from the time domain, frequency domain, and energy domain to compensate for the insufficiency of single-indicator discriminative capability, and sequentially achieves qualitative identification, spatial localization, and quantitative assessment of damage severity. To further standardize the diagnostic procedure, three standardized indicators—spectral distortion, defect localization, and damage evolution—are introduced, each with clear physical meanings. These three indicators form a complementary graded diagnostic criterion of “qualitative–localization–quantitative”, providing a unified quantitative framework for graded evaluation of the health state of rock bolts.

Dominant Frequency Drift Rate (R_f_): The dominant frequency drift rate quantifies the deviation of the spectral main peak in the defective condition from the defect-free baseline, characterizing the spectral mutation features. It is particularly suitable for identifying the frequency multiplication phenomenon at the minor defect stage [31], and is defined as:

(5) $$R_{f}=\frac{\left|f_{p}-f_{0}\right|}{f_{0}}\times 100\%$$

where f_0_ is the reference dominant frequency in the defect-free state (approximately 3 MHz), and f_p_ is the dominant frequency extracted under defective conditions. A larger R_f_ indicates more significant spectral distortion, which can serve as a qualitative screening indicator for minor defects.

Echo Time Delay Indicator (Δt): This indicator reflects the variation in propagation travel time induced by defects and is used for defect localization analysis. It is defined as follows:

(6) $$\Delta t=t_{0}-t_{d}$$

where t_0_ is the arrival time of the first echo from the bottom end under defect-free conditions, and t_d_ is the arrival time of the first echo reflected from the defect under defective conditions. Since the defect induces local reflection and disturbance to the propagation path, the arrival time of the echo shifts accordingly; therefore, Δt can be used to estimate the defect location. Under ideal homogeneous medium conditions, the position of the defect relative to the bottom end of the bolt x can be estimated from this time delay as follows:

(7) $$x\approx \frac{v\Delta t}{2}$$

where v is the P-wave propagation velocity in the bolt material (approximately 5900 m/s). This indicator provides a reference for identifying the spatial distribution of defects in engineering applications.

Energy Attenuation Coefficient (R_E_): The energy attenuation coefficient quantifies the degree of signal energy loss caused by defects and serves as a core indicator for damage severity assessment. It is defined as follows:

(8) $$R_{E}=\frac{E_{0}-E_{d}}{E_{0}}\times 100\%\;$$

where E_0_ is the received signal energy in the defect-free reference state, and E_d_ the signal energy under defective conditions. The simulation results of this study indicate that the energy indicator is the most sensitive to variations in defect size among all characteristic parameters, and can stably reflect the enhanced scattering and dissipation effects induced by defects. Therefore, RE can serve as the master control parameter for quantitative damage assessment and health grading.

Based on the above three indicators and using the defect-free rock bolt as the benchmark, this study establishes a health grading standard for deep anchoring structures (Table 7), incorporating the principles of dominant frequency multiplication and nonlinear energy attenuation. This standard classifies the anchorage quality into four grades (I–IV): healthy, mild, moderate, and severe. The dominant frequency drift rate serves for qualitative early warning, the echo time delay for defect localization, and the energy attenuation coefficient for quantitative grading.

To verify the reliability of the simulation results, the defect response characteristics obtained in this study are compared with existing findings in the literature. Guo et al. [32] reported that the defect size does not affect the arrival time of the echo but primarily influences the amplitude. In the present study, the arrival time of the first wave at the lateral monitoring point remains stable at 2.03 μs, while the amplitude decreases with increasing defect size, which is consistent with their findings. Li et al. [33] demonstrated experimentally that the transmitted energy exhibits a significant decreasing trend as the defect severity increases. The energy attenuation rates obtained in this study, ranging from 21% to 61%, are in good agreement with this trend. Further analysis of the correlation between frequency-domain parameters and anchorage quality reveals that the dominant frequency at the main-axis point exhibits a frequency multiplication transition (2.99–6.18 MHz), whereas that at the lateral point is confined to a lower frequency band (5.98–2.99 MHz). The frequency response discrepancy between the two monitoring points highlights the sensitivity of high-frequency components to the identification of minor defects and indicates that the dominant frequency drift feature outperforms conventional time-domain parameters in defect characterization. The thresholds listed in Table 7 are derived from the present simulation cases and serve as preliminary reference values, providing a basis for further calibration and extension with additional data in future work.

## 5. Conclusions

To address the issues of durability deterioration and health diagnosis methodology for anchoring structures, this study employs theoretical analysis, multi-physical field simulation, and ultrasonic nondestructive testing (NDT) simulation approaches to reveal the intrinsic relationship between temperature-dependent corrosion effects and ultrasonic response characteristics. The main conclusions are as follows:

(1) Temperature exerts a significant accelerating effect on the electrochemical corrosion of rock bolts. In the range of 283 K–323 K, the corrosion rate increases from 1.577 mm/a to 2.315 mm/a, representing an increase of 46.8%. The rise in temperature shifts the equilibrium potential negatively and causes an exponential increase in the reaction rate constant, which synergistically enhances anodic dissolution. The axial potential distribution along the bolt exhibits a “higher in the middle, lower at both ends” pattern, and the current density at the bolt ends is approximately 1.8 times that in the middle section. The end effect makes the two ends the priority corrosion zones.

(2) The modulation effect of defects on ultrasonic signals exhibits a significant spatial dependence, and the damage information reflected by different monitoring locations is complementary. The main-axis monitoring point is located in the transmission-focused zone, where acoustic waves undergo symmetric diffraction and in-phase superposition upon encountering defects, causing minor defects to induce dominant frequency multiplication. The lateral monitoring point is situated in the reflection–interference zone, where the signal is formed by the superposition of the direct wave and the reflected wave from the defect. For minor defects, the waveform shows no significant change, but energy attenuation occurs; for major defects, the first wave is delayed, the amplitude decreases, and the energy is substantially attenuated.

(3) Based on the spatial complementary mechanism, a four-level grading diagnosis standard is proposed with the dominant frequency drift rate, echo time delay, and energy attenuation coefficient as the core indicators. A dominant frequency drift rate exceeding 50% is suggested as the early warning threshold for minor defects, while an energy attenuation coefficient exceeding 50% accompanied by an echo time delay greater than 0.5 μs is defined as the simulation-based threshold for macroscopic fracture identification.

## Figures and Tables

**Table 2:** Table of values for anchor materials.

Properties	Value	Units
Density ρ	7850	kg/m^3^
Elasticity modulus E	200	GPa
Poisson ratio ν	0.30	—
Conductivity k	4.032 × 10^6^	S/m
Longitudinal wave velocity $v_{\mathrm{Fe}}$	5900	m/s

**Table 3:** Comparison of theoretical and observed values of ultrasonic time delay for different propagation segments.

Segment Number	Geometrical Distance	d (m)	Theoretical Time Delay T (μs)	Observation Delay ∆tobs (μs)	Relative Error (%)
1	6.10	0.006100	1.033898	1.050000	1.56
2	7.28	0.007280	1.233898	1.250000	1.30
3	7.28	0.007280	1.233898	1.250000	1.30

**Table 4:** Comparison of ultrasonic signal characteristic parameters under different defect sizes (Collection Points: 15, 0).

Characteristic Parameter	Defect-Free Model	1 × 1 Defect	2 × 2 Defect	3 × 3 Defect
First wave arrival time (μs)	3.47	3.4	3.4	4.28
Propagation delay (μs)	0	−0.07	−0.07	0.82
Main peak amplitude (Pa)	1.08 × 10^6^	1.38 × 10^6^	1.38 × 10^6^	1.99 × 10^6^
Signal energy (Pa^2^·s)	5.13 × 10^5^	7.96 × 10^5^	7.96 × 10^5^	2.68 × 10^6^
Dominant frequency (MHz)	2.99	6.18	6.18	2.99

**Table 5:** Comparison of ultrasonic signal characteristic parameters under different defect sizes (Collection Points: 20, 10).

Characteristic Parameter	Defect-Free Model	1 × 1 Defect	2 × 2 Defect	3 × 3 Defect
First wave arrival time (μs)	2.05	2.03	2.03	2.92
Propagation delay (μs)	0	−0.02	−0.02	0.87
Main peak amplitude (Pa)	1.14 × 10^7^	1.11 × 10^7^	1.11 × 10^7^	4.36 × 10^6^
Signal energy (Pa^2^·s)	5.46 × 10^7^	4.30 × 10^7^	4.30 × 10^7^	2.11 × 10^7^
Dominant frequency (MHz)	5.98	2.99	2.99	2.99

**Table 6:** Evaluation table of response characteristics of multi-feature parameters and matching table of diagnostic strategies.

Fault location Monitor	Characteristic Parameter	Minor Defects (1–2 mm)	Large-Sized Defect (3mm)	Diagnostic Assessment
Main axis line monitoring points	dominant frequency	Double (+106%)	uniformity	Qualitative early warning
Main axis line monitoring points	Amplitude/energy	Significantly enhanced (+28%/+55%)	Violent eruptions (+84%/+420%)	assisting determination
Lateral monitoring point	signal energy	Significant attenuation (−21%)	Severe attenuation (−61%)	quantitative evaluation
Lateral monitoring point	propagation time delay	No significant change (<0.05 μs)	Significant lag (+0.87 μs)	Severe diagnosis

**Table 7:** Grading criteria for the health status of deep anchoring structures.

Health Level	Faulted Condition	Main Frequency Drift Rate (R_f_)	Energy Attenuation Coefficient (R_E_)	Echo Time Difference (Dt)	Disposal Suggestions
Level I	Health (Complete)	<5%	<10%	There are no obvious defect echoes	Maintain regular monitoring during normal use
Level II	Mild injury (Micro fissures)	>50%	10~30%	The first wave showed no significant deviation	Encrypted monitoring frequency
Level III	moderate injury (Expansive cracks/Corrosion)	Messy or high-frequency truncation	30~50%	Weak defect waves can be observed	Conduct a pull force check
Level IV	Severe injury (Through fracture)	Irregular or low-frequency locking	>50%	Significant lag/independent echo	Reapply rock bolts or replace support components

## Data Availability

The original contributions presented in this study are included in the article. Further inquiries can be directed to the corresponding authors.
